# Dielectric and Biological Characterization of Liver Tissue in a High-Fat Diet Mouse Model

**DOI:** 10.3390/s23073434

**Published:** 2023-03-24

**Authors:** Clément Buisson, Lourdes Mounien, Flavie Sicard, Jean-François Landrier, Victoria Tishkova, Pierre Sabouroux

**Affiliations:** 1 Aix Marseille Univ, CNRS, Centrale Marseille, Institut Fresnel, Marseille, France; 2 Aix Marseille Univ, CNRS, CINaM, Marseille, France; 3 Aix-Marseille Université, C2VN, INRAE, INSERM, Marseille, France; 4 PhenoMARS Aix-Marseille Technology Platform, CriBiom, Marseille, France

**Keywords:** dielectric permittivity, *ex vivo* tissues, fatty and healthy liver, triglyceride

## Abstract

Hepatic steatosis may be caused by type 2 diabetes or obesity and is one of the origins of chronic liver disease. A non-invasive technique based on microwave propagation can be a good solution to monitor hepatic tissue pathologies. The present work is devoted to the dielectric permittivity measurements in healthy and fatty liver in the microwave range. A mouse model following normal and high sugar/glucose (HFS) diets was used. We demonstrated the change in the triglyceride and glucose concentration in the hepatic tissue of HFS diet mice. The difference in the dielectric permittivity of healthy and fatty liver was observed in the range from 100 MHz to 2 GHz. The dielectric permittivity was found to be 42 in the healthy tissue and 31 in the fatty liver tissue at 1 GHz. The obtained results demonstrate that dielectric permittivity can be a sensitive tool to distinguish between healthy and fatty hepatic tissue.

## 1. Introduction

The interaction of a biological tissue with an electromagnetic wave is determined by the dielectric properties of the tissue, namely conductivity and relative permittivity. These two quantities are complex and can be described empirically with the Debye model [1]. The dielectric characterization of biological tissues is performed more and more frequently in medical diagnostics and therapeutics [2,3] and is commonly used for tumor ablation [4]. Another application is the safety assessment of microwave exposure; the specific absorption rate (SAR) can be calculated only if the complex relative permittivity of a tissue is known [5]. All the future medical applications of microwaves are based on dielectric properties and accurate measurements. Several research groups have been working on the subject. The most known work [6] published research on the relative permittivity and conductivity of major bovine and porcine tissues, such as skin, liver, or lung, at frequencies from 10 Hz to 20 GHz. Other groups have focused on the characterization of healthy and malignant tissues [7,8]. Comparative analyses of the dielectric properties of breast tissue and tumor tissue were reported by [8,9]. It was also shown that microwave detection of intracranial hemorrhage is promising due to the change in the dielectric properties [10]. Moreover, non-invasive blood glucose detection was shown to be possible due to the variation in the blood permittivity with glucose concentration [11,12,13].

Non-alcoholic fatty liver disease (NAFLD) is a spectrum of liver abnormalities occurring without excess alcohol consumption. It includes hepatic steatosis, fibrosis, cirrhosis, and hepatocellular carcinoma [14]. According to World Health Organization, NAFLD has become the most common cause of chronic liver disease [15]. The present work focuses on hepatic steatosis, which may be mainly caused by type 2 diabetes or obesity. In the case of hepatic steatosis, lipids, mainly triglycerides, are stored in the liver between hepatic cells. Imaging techniques such as ultrasound, computerized tomography, and magnetic resonance imaging are used to diagnose hepatic steatosis but, unfortunately, are rather expensive, and the sensitivity of these techniques is limited. Recently, a method for the detection of hepatic steatosis based on microwave propagation was proposed [16,17,18]. This technique is based on the difference in the dielectric permittivity of healthy and fatty liver. In the literature, one can find measurements of the dielectric characterization of healthy and diseased liver. Changes in the dielectric permittivity of normal, malignant, and cirrhotic liver tissue *in-vivo* and *ex-vivo* were studied in human and porcine liver [9,19]. Several works are devoted to the influence of hydration on the dielectric permittivity of the liver tissue [20,21,22]. Peyman and al. [19] noticed a difference in hepatic dielectric permittivity in the case of a patient with steatosis compared to healthy tissue, but to the best of our knowledge, there is no systematic measurements of the dielectric permittivity as a function of liver steatosis. It was shown experimentally in rats and mice that heavily based sucrose/glucose diets cause an increase in the weight of the animals [23,24] as well as in some organs, notably the liver [25]. Indeed, there is an important increase of hepatic triglycerides/glycemia and plasmatic triglycerides/glycemia for animals under a sucrose/glucose diet [23,24,25,26,27].

The main goal of the present work is to study the dielectric permittivity contrast between healthy and fatty liver. To measure the permittivity, we use a technique that is generally not, or very rarely, used for this type of biological material: the coaxial propagation line technique. This technique was chosen due to the small sample volume available for the measurements. We analyze real and imaginary parts of the permittivity of mouse liver on normal (control) and heavily-based sucrose/glucose diets. Finally, we compare our results with those of the literature.

## 2. Materials and Methods

### 2.1. Animals, Diets, and Protocol

For this experiment on mice, the protocol received the agreement of the local ethics committee and the French Minister of Research and Education (ethical approval number n°27998-201909241709755 v9). Six-week-old male C57BL/6J mice were obtained from Janvier Labs (Le Genest Saint Isle, France) and fed ad libitum with control food (chow diet A04 from Safe-diets) during a 1 week acclimation period, with full access to drinking water. The animals were maintained at 22 °C under a 12 h light, 12 h dark cycle at 20% humidity. Mice (10 per group) were randomly assigned into one of the two experimental groups depending on the diet, that is, control (chow diet AIN-93G from Safe diets) or high fat (HF: 60% energy from lipids, Test Diet ref. 58V8). Twelve weeks after the beginning of the protocol, the mice fasted overnight. Blood sampling was performed by cardiac puncture under general anesthesia. Plasma was prepared by centrifugation at 3000 rpm for 15 min at 4 °C and was stored at −80 °C. Mice were euthanized by cervical dislocation under general anesthesia, and the livers were collected, weighed, and used for dielectric parameters measurements. The time between the liver excision and dielectric measurements was 3 to 6 min.

To perform a comparative analysis of our set-up for dielectric measurements and the commonly used open-ended coaxial probe technique, lamb liver was used. It was obtained from freshly slaughtered juvenile (1 year old) male cattle. The liver was transported to the laboratory in ice, where the dielectric parameters measurements were performed.

### 2.2. Biochemical Analysis

All parameters were quantified as previously reported, such as in [24,25,26]. Briefly, glucose concentrations in plasma and liver were evaluated using glucose RTU (bioMerieux, Craponne, France). Triglycerides (TG) were measured by colorimetric methods (RANDOX, Crumlin, Co., Antrim, UK).

### 2.3. Dielectric Analysis

Currently, most of the measurements of dielectric parameters made on biological tissues use the open-ended coaxial probe technique [3,9,28,29]. However, for our experimental protocol, the amount of liver tissue in the mice was not sufficient to use the open-ended coaxial probe. Therefore, we opted for a different system, using a coaxial transmission line. This technique was more convenient for these tissues since it requires a smaller volume of material under test. Moreover, it allows for measurement of the mean dielectric properties for the whole volume of the sample and not only the surface. Coupled with this coaxial cell, we used a Vector Network Analyzer (VNA) Anritsu MS2036C [30].

**Figure 1:**
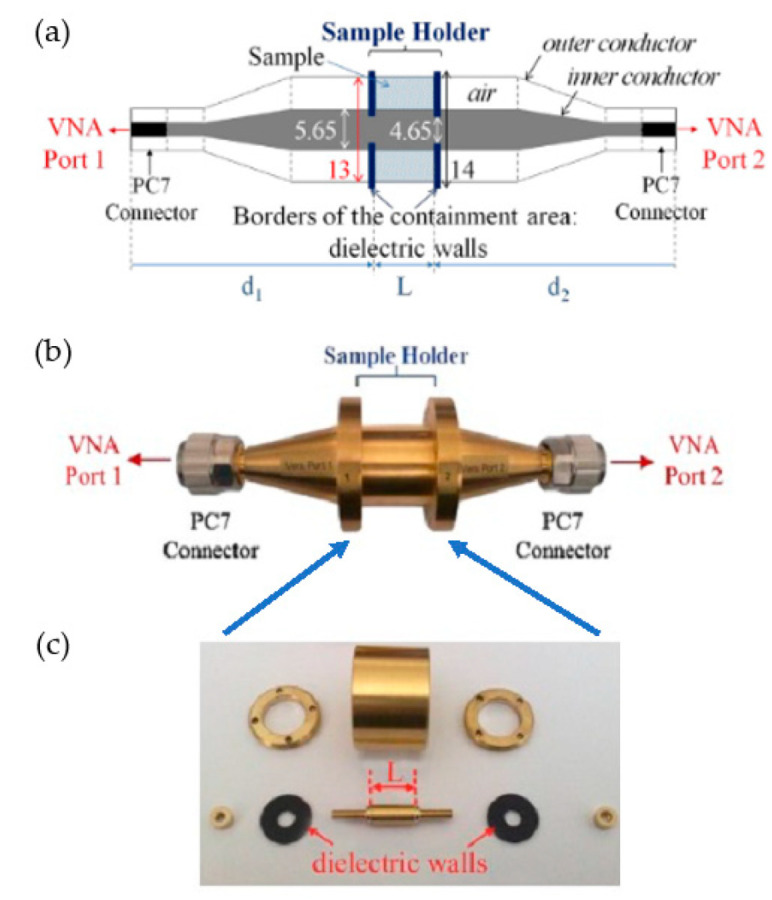
(**a**) Schematic diagram of the coaxial cell. (**b**) Photo of the fully assembled coaxial cell. (**c**) Dismantled coaxial cell.

The major parts of the measuring coaxial cell used in our experiments are represented in Figure 1. The schematic view in Figure 1a illustrates the coaxial cell used, the photo in Figure 1b shows the mounted cell, and Figure 1c shows the dismantled cell with all the parts of the sample holder. The length L = 3 mm of the sample holder, and the distance d_1_ = d_2_ = 96 mm. The total volume of the sample holder is 0.7 cm^3^ [31]. Before the sample measurements, a calibration procedure must be performed. The first step is the “open, short, load, thru” (OSLT) calibration of the VNA. In the second step, the coaxial measuring cell must be calibrated with a short circuit component and a simple empty coaxial line.

**Figure 2:**
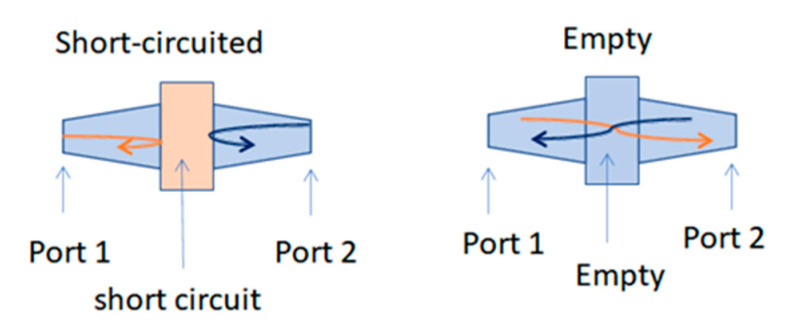
Description of the protocol measurements. (The orange arrow is forward propagation, and the blue one is reverse propagation).

The second step of the measurement protocol is schematically described in Figure 2. The arrows in Figure 2 represent two electromagnetic waves propagating in the measuring coaxial cell. In the case of the short circuit, both waves are totally reflected, which allows for the measurement of the reflection coefficients. In the case of the empty cell, the total transmission allows the reference measurement of transmission coefficients. These coefficients, more commonly known as the S matrix, are complex values and are measured with the VNA.

(1) $$S_{matrix}=\left(\begin{array}{cc} S_{11} & S_{12}\\ S_{21} & S_{22} \end{array}\right)$$

Visualization of a *S* matrix is in Equation (1). The numbers after the letter translate to the reception port and the transmission port of the electromagnetic wave.

S_11_ port 1 emitter and port 1 receiver (reflection coefficient)
S_21_ port 1 emitter and port 2 receiver (transmission coefficient)
S_12_ port 2 emitter and port 1 receiver (transmission coefficient)
S_22_ port 2 emitter and port 2 receiver (reflection coefficient)

The third step is to perform measurements of the sample to obtain the S-matrix called measurement. The last step is to determine the S-matrix of the sample using the previous S matrixes (S matrix of the short circuit, empty cell, and the measurement). To do this, a de-embedding operation is applied [32]. The de-embedding operation is described with the following expressions [33]:

(2) $${\left|S_{ii}\right|}_{s}=\frac{{\left|S_{ii}\right|}_{m}}{{\left|S_{ii}\right|}_{sc}}\;{\left|S_{ij}\right|}_{s}=\frac{{\left|S_{ij}\right|}_{m}}{{\left|S_{ij}\right|}_{em}}\;\varphi_{ii}=\varphi_{iim}-\varphi_{iisc}-\pi \;\varphi_{ij}=\varphi_{ijm}-\varphi_{ijem}-kL$$

With:

(*j*,*j*) ∈ [1;2]
*k*: wave vector
*L*: length sample size
*ω*: Angular frequency
*s*: sample
*m*: measurement
*sc*: short-circuit
*em*: empty

Equation where │*S_ii/ij_*│ and $\varphi_{ii/ij}$ translate respectively to the module and phase of the S-parameter [32] is visualize in Equation (2).

From the S matrix of the sample, we calculate the values of the complex permittivity of the liver sample using the Nicolson-Ross calculation protocol. The main relations described by Nicolson-Ross are written below [34]:

(3) $$\varepsilon =i\sqrt{\frac{\varepsilon_{0}}{\mu_{0}}}\frac{ln(T)}{\omega L}\frac{1-\Gamma }{1+\Gamma }\;\Gamma =\frac{\left(S_{11}^{2}-S_{21}^{2}+1\right)-C}{2\;S_{11}}\;T=\frac{\left(S_{21}^{2}-S_{11}^{2}+1\right)-C}{2\;S_{21}}\;C=\sqrt{\left(S_{11}+S_{21}+1)(S_{11}+S_{21}-1)(S_{11}-S_{21}+1)(S_{11}-S_{21}-1\right)}$$

Equation (3) are the Nicolson-Ross equations, where Γ, *T*, and *C* are intermediate variables used for the *S* parameters calculations and are explained in [30].

**Figure 3:**
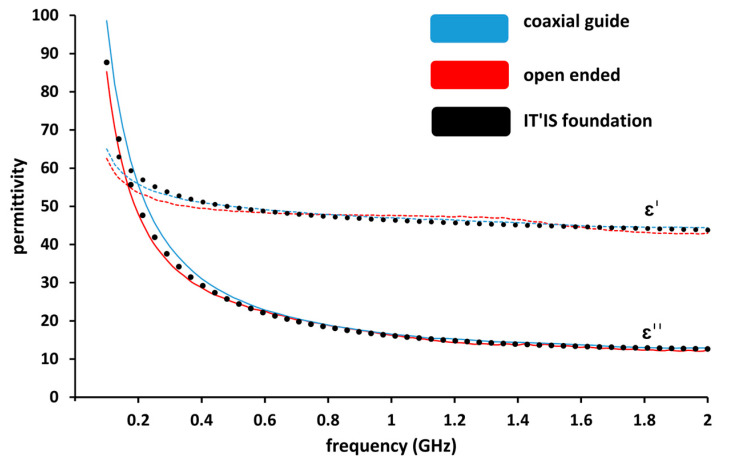
Comparison of real part (ε′) and imaginary part (ε″) permittivity measurements of lamb liver using a coaxial guide and an open-ended probe (SPEAG DAK 3.5) as a function of frequency.

To validate the coaxial propagation line technique on biological tissue, we compared measured data with the open-ended probe technique. The used model in the lab is a SPEAG DAK3.5 open-ended coaxial probe. For this purpose, we used lamb liver which has sufficient volume. It should be noticed that the dielectric permittivity of the measured lamb liver tissue is similar to the dielectric permittivity of human liver, according to Gabriel et al., 1996 [6]. We therefore added the dielectric properties of human liver provided by the IT’IS Foundation [35] in Figure 3.

The measurements of lamb liver *ex-vivo* tissue were performed over the frequency range 100 MHz–2 GHz and are presented in Figure 3. The obtained result proves the repeatability of the measurements for both used techniques as the results measured with both techniques are superposed. We conclude that the coaxial line measurement technique is as reliable as the open-ended measurement technique.

### 2.4. Statistical Analysis

Values were reported as mean ± s.e.m. All data were successfully tested for normality using a Shapiro-Wilk test and then analyzed with a two-tailed, unpaired *t*-test. Statistical analyses were performed with Prism v9 (GraphPad 9.3.1 Software). A *p* value inferior to 0.05 indicated statistical significance.

## 3. Results

**Figure 4:**
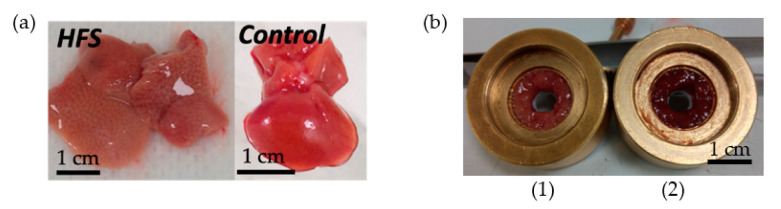
*Ex vivo* mice liver samples. (**a**) An HFS Diet liver sample and control diet liver sample. (**b**) Samples of liver in the sample holder (0.32 cm^3^): on the left HFS Diet liver (1) and on the right control Diet liver (2).

**Figure 5:**
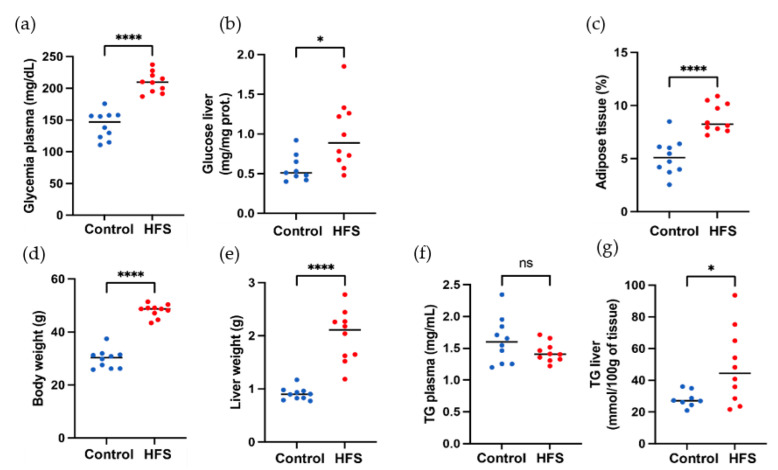
Morphological and biochemical parameters of mice. (**a**) Body weight, (**b**) liver weight, (**c**) adiposity, (**d**) glycemia plasma level in liver, (**e**) glucose level in liver, (**f**) TG plasma liver, and (**g**) TG liver. Red color corresponds to HFS diet mice, and blue color to control mice, ns: *p*-value > 0.05; *: *p*-value ≤ 0.05; ****: *p*-value ≤ 0.0001.

The effect of a high-fat diet (60% energy from fat, HFS diet group) compared to a control diet (AIN control group) was evaluated in WT C57BL/6J male mice for 12 weeks. Some samples from the HFS diet and control diet are visible in Figure 4. Body weight and liver weight increased in the HFS diet group Figure 5D,E. Adiposity weight (sum of epididymal, inguinal, and retroperitoneal adipose tissue mass) increased in the HFS diet group Figure 5C. The HFS diet led to increased glycemia and glucose levels in the liver Figure 5A,B. The TG levels in plasma were similar in control and HFS diet mice, but the TG content in the liver increased in the HFS diet group compared to control animals. The level of TG and glucose in the liver did not allow us to distinguish between the two groups and to conclude on the state diet of an animal.

**Figure 6:**
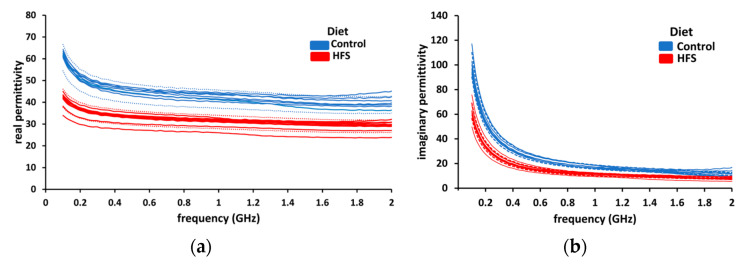
The dielectric permittivity of liver samples. (**a**) The real part of permittivity (blue line) of control diet liver samples and the real part of permittivity of HFS diet liver samples (red lines). (**b**) The imaginary part of permittivity (blue lines) of AIN diet liver samples and the imaginary part of permittivity of HFS diet liver samples (red lines).

During the dielectric measurement campaign, six samples of control group liver and ten samples of HFS diet liver were tested. The real and imaginary parts of permittivity are plotted together in Figure 6. Two colors will be used to distinguish between healthy (control AIN diet-blue) and fatty (HFS diet-red) liver throughout this manuscript. To show the reproducibility of the measurements, we set a confidence range of ±10% (dotted lines) around the mean value for both groups. In the case of HFS diet liver, there is a noticeable diminution of real and imaginary parts of permittivity compared to the control group. (Real and imaginary parts of permittivity have diminutions of 25 and 35%, respectively.) These measurements show that it is possible to distinguish, in *ex-vivo,* a healthy liver from an HFS-diet-fed mouse liver based on the dielectric permittivity measurements.

**Figure 7:**
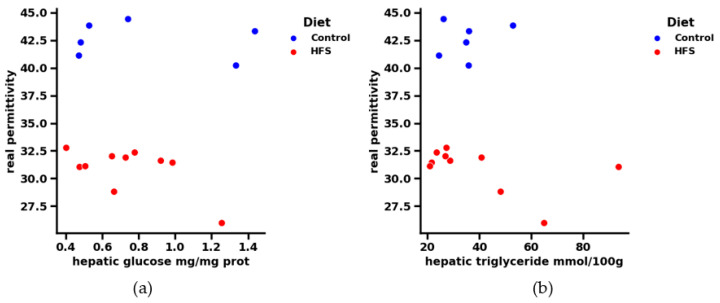
(**a**) Correlation between real permittivity and liver tissue glucose at 1 GHz. The regime is dissociated by color: blue for the control and orange for the HFS diet. (**b**) Correlation between real permittivity and hepatic triglyceride of liver tissue at 1 GHz. The regime is dissociated by color: blue for the control and orange for the HFS diet.

For the following analysis, we chose to represent only 1 GHz, as the results remain constant with the frequency: Figure 7 shows the real part of permittivity in the function of hepatic triglycerides and glycemia, respectively, at a frequency 1 GHz. Contrary to hepatic TG and glucose, the real part of the dielectric permittivity seems to differentiate between healthy and HFS diets. Indeed, if we look at the range [0.4–0.6] (mg/mg protein) for hepatic glycemia and the range [20–40] mmol/100 g for hepatic triglycerides, we see that if the diet followed by the subjects is unknown, it is no longer possible to distinguish between them using only the hepatic physiological parameters. The contribution of the dielectric parameters thus makes it possible to highlight the type of diet followed by the mice. Indeed, some physiological points very close to each other can be dissociated by knowing the permittivity of each point. It is therefore easy to know which mouse followed which regime, according to the correlation of these data. Knowledge of the dielectric permittivity of liver tissue complements the biological data and supports the application of a microwave testing technique.

## 4. Discussion

Dielectric properties of hepatic tissues from recent literature and the present study are summarized in Table 1.

One observes that *ex-vivo* healthy hepatic dielectric permittivity from the present work is lower than the *in-vivo* measurements [19,36,37]. This correlates with the comparison of the *ex-vivo* and *in-vivo* data observed in [36], where authors showed that the *ex-vivo* hepatic dielectric permittivity decreases with time, and the major difference is observed 4–6 min after excision. Healthy tissue real permittivity shows different values for each animal model at 1 GHz. In the case of rat tissue, authors found real permittivity values to be 49 [36] and 47 [37]. Similar results are obtained for porcine 50 [20] and bovine tissue 49 [21] and 46 [20]. Human healthy hepatic tissue real permittivity is 46 [19]. Results from our work show lower mean real permittivity (42 ± 2) of healthy tissue compared to the literature. To the best of our knowledge, our results are the only results presenting measurements of hepatic dielectric properties on a mouse model. At the same time, all the experimental results from Table 1 were performed by an open-ended probe. We suppose that this difference originates from the animal model we used as an open-ended coaxial probe that showed the same measured values on a test liver sample. Malignant and cirrhosis liver tissues have higher dielectric permittivities than healthy tissue [19,37]. In the case of fatty liver, only one study measured the dielectric permittivity of *ex-vivo* human hepatic steatosis [19]. In this study, authors observed a decrease in hepatic steatosis dielectric permittivity. In the present work, we also observed a decrease in dielectric permittivity for the HFS diet mouse liver contrary to control group. Analysis of the results shows that healthy, fatty, malignant, and cirrhosis liver can be differentiated by dielectric permittivity measurements. This result is promising for the application of microwave monitoring of liver pathologies.

## 5. Conclusions

Hepatic steatosis, an accumulation of TG in the liver, is often observed in patients with obesity and type 2 diabetes. It was shown previously that in rats and mice, a sucrose/glucose diet leads to the accumulation of hepatic TG. We used an animal model to investigate changes in the hepatic tissue dielectric properties caused by an HFS diet. A coaxial transmission line was used to characterize the dielectric permittivity of tissues. When tested, this technique used a smaller volume of material than an open-ended coaxial probe. Moreover, the mean dielectric permittivity of the whole sample and not only the surface was measured.

Structural and chemical modifications of hepatic tissues were observed: hepatic and plasmatic glucose and TG increased due to an HFS diet, but due to dispersion of the measured parameters, it was not possible to distinguish between hepatic tissue of control and HFS diet mice. Interestingly, the dielectric permittivity of the hepatic tissue was measured in the range of 100 MHz–2 GHz, and it was observed to decrease for the mice following an HFS diet (fatty liver). The literature shows that, in general, there is a dielectric contrast between healthy and diseased liver, and the experimental results in this study were found to be in agreement with the literature.

## Figures and Tables

**Table 1:** Dielectric properties of hepatic tissues from recent literature and the present study.

Reference	Type of Measurement	Species	Liver Tissue	Frequency Range	Real Permittivity (1 GHz)
Peyman and all 2015 [19]	*ex-vivo* (1 h max)	human	Healthy	200 MHz–5 GHz	46
			Tumor		42
			Steatosis		44
Farrugia and all 2016 [36]	*in-vivo*	rat	Healthy	500 MHz–40 GHz	49
	*ex-vivo* (1 min)				52
	*ex-vivo* (6 min)				49
Shahzad and all 2017 [22]	*ex-vivo* (8 ± 3 min)	bovine	Healthy	500 MHz–20 GHz	48
Yilmaz 2020 [37]	*in-vivo*	rat	Healthy	500 MHz–6 GHz	47
			Malignant		55
			cirrhosis		53
Shahzad et al., 2020 [21]	*ex-vivo*	bovine	Healthy	500 MHz–8.5 GHz	49
Di Meo et al., 2021 [20]	*ex-vivo*	porcine		0.5 GHz–50 GHz	50
		ovine			48
		bovine			46
		human cadaver			52
Present study	*ex-vivo* (3–6 min)	mouse	Healthy	100 MHz–2 GHz	42 ± σ = 2
			HFS diet		31 ± σ = 3 σ: deviation
